# Transcriptome and proteome profiling of activated cardiac fibroblasts supports target prioritization in cardiac fibrosis

**DOI:** 10.3389/fcvm.2022.1015473

**Published:** 2022-12-01

**Authors:** Maria Raquel Moita, Marta M. Silva, Cláudia Diniz, Margarida Serra, René M. Hoet, Ana Barbas, Daniel Simão

**Affiliations:** ^1^iBET - Instituto de Biologia Experimental e Tecnológica, Oeiras, Portugal; ^2^Instituto de Tecnologia Química e Biológica António Xavier, Universidade NOVA de Lisboa, Oeiras, Portugal; ^3^Department of Pathology, CARIM - School of Cardiovascular Diseases, Maastricht University, Maastricht, Netherlands; ^4^Bayer Portugal, Carnaxide, Portugal

**Keywords:** cardiac fibroblast, cardiac fibrosis, myofibroblast, transcriptomic, quantitative proteomics

## Abstract

**Background:**

Activated cardiac fibroblasts (CF) play a central role in cardiac fibrosis, a condition associated with most cardiovascular diseases. Conversion of quiescent into activated CF sustains heart integrity upon injury. However, permanence of CF in active state inflicts deleterious heart function effects. Mechanisms underlying this cell state conversion are still not fully disclosed, contributing to a limited target space and lack of effective anti-fibrotic therapies.

**Materials and methods:**

To prioritize targets for drug development, we studied CF remodeling upon activation at transcriptomic and proteomic levels, using three different cell sources: primary adult CF (aHCF), primary fetal CF (fHCF), and induced pluripotent stem cells derived CF (hiPSC-CF).

**Results:**

All cell sources showed a convergent response upon activation, with clear morphological and molecular remodeling associated with cell-cell and cell-matrix interactions. Quantitative proteomic analysis identified known cardiac fibrosis markers, such as FN1, CCN2, and Serpine1, but also revealed targets not previously associated with this condition, including MRC2, IGFBP7, and NT5DC2.

**Conclusion:**

Exploring such targets to modulate CF phenotype represents a valuable opportunity for development of anti-fibrotic therapies. Also, we demonstrate that hiPSC-CF is a suitable cell source for preclinical research, displaying significantly lower basal activation level relative to primary cells, while being able to elicit a convergent response upon stimuli.

## Introduction

Cardiac fibrosis is implicated in almost all forms of cardiovascular pathologies, negatively impacting disease progression and clinical outcomes (1, 2). This condition is characterized by excessive deposition of extracellular matrix (ECM) proteins which leads to scar formation and impairment of cardiac function (3). As for many fibrotic conditions, effective therapies to treat cardiac fibrosis are still not available (4–6).

Cardiac fibroblasts (CF) are key cellular effectors on the development of cardiac fibrosis (1, 7). When quiescent CF convert into activated CF, also known as myofibroblasts, these cells acquire a highly proliferative and secretory phenotype, producing excessive amounts of ECM proteins responsible for limiting cardiac function (8, 9). Myofibroblasts can originate from different cell sources, including fibroblasts, mesenchymal stem cells, endothelial cells, and immune cells (10, 11). However, in the heart, the main source of myofibroblasts are CF (12, 13). Despite the substantial progress in our understanding of CF activation mechanisms, the number of actionable targets in cardiac fibrosis remains limited. The lack of sustainable sources of CF and reliable cell culture methods have been hampering the identification of putative targets and drug development studies (14–17).

To address this translational gap, we investigated the transcriptome and proteome remodeling of three cell sources of human CF upon activation: adult primary CF (aHCF), fetal primary CF (fHCF), and CF-derived from induced pluripotent stem cells (hiPSC-CF). Prior to activation with TGF-β1, all CF sources were cultured under conditions that allowed maintenance of the quiescent state. CF treatment with TGF-β1 resulted in clear phenotypic alterations in all CF sources which are in accordance with hallmarks of CF activation (9, 18). Transcriptome remodeling revealed a convergent response of all CF by modulation of biological processes related to adhesion, extracellular matrix organization, cell migration, and angiogenesis. At the proteome level we identified proteins already linked to fibrotic conditions but not previously associated with fibrosis. Thereby we provide a panel of putative novel targets for cardiac fibrosis. In addition, we demonstrate that hiPSC-CF is a suitable and sustainable cell source for *in vitro* drug discovery displaying a significantly lower basal activation level as compared with primary cells, while retaining the ability to elicit a convergent response relative to primary cells upon stimuli.

## Materials and methods

### Differentiation of human-induced pluripotent stem cells into quiescent cardiac fibroblasts

Human-induced pluripotent stem cells (hiPSCs), wild-type background C (WTC, UCSFi001-A) (19), were differentiated into cardiac fibroblasts based on previous reports (20, 21). Briefly, hiPSCs were propagated on surfaces coated with growth factor reduced phenol-red free Matrigel (BD Biosciences) in mTeSR1™ medium (STEMCELL Technologies, Saint Égrève, France). At 80–90% of confluence, cells were passaged using Accutase (STEMCELL Technologies, Saint Égrève, France), seeded at 2–3 × 10^4^ cell/cm^2^, and cultured on mTeSR1™ medium supplemented with 10 μM ROCK-specific inhibitor (Y-27632; TOCRIS, United Kingdom). To start the differentiation hiPSCs were seeded at 2–3 × 10^5^ cell/cm^2^ on 12-well plates. The first stage of differentiation, from hiPSCs into Cardiac Progenitor Cells (CPCs), was initiated 3 days after (day 0) by replacing the culture medium with RPMI 1640 basal medium (Thermo Fisher Scientific, USA) supplemented with 6 μM CHIR99021 (TOCRIS, United Kingdom). On day 1, the media was aspirated and replaced by RPMI 1640 basal medium. On day 3, the media was exchanged by a “combined medium” comprising of 1 mL of the spent media and 1 mL of freshly prepared RPMI 1640 basal medium supplemented with 2.5 μM IWP-2 (TOCRIS, United Kingdom), per well. On day 5, the medium was replaced with RPMI 1640 medium. On day 6, the second stage of differentiation from CPCs to pro-epicardial cells started. Human-induced pluripotent stem cell-CPCs were seeded at a density of 8 × 10^4^ cell/cm^2^ on Synthemax II-SC (Corning^®^, USA)-coated 12-well plates and cultured in LaSR medium [Advanced DMEM/F12 medium (Thermo Fisher Scientific, USA), supplemented with GlutaMAX™ (Thermo Fisher Scientific, USA), 100 mg/mL Ascorbic Acid (Sigma-Aldrich, USA), and 5 μM Y-27632 (TOCRIS, United Kingdom)]. From day 7 to day 8, medium was replaced by LaSR medium supplemented with 3 μM CHIR99021. From day 9 to day 11, medium was exchanged daily by LaSR medium. The last stage of differentiation, from pro-epicardial cells into cardiac fibroblasts (CF) started on day 12. Cells were seeded at a density of 3 × 10^4^ cell/cm^2^ on 0.1% (w/v) gelatin-coated (Sigma-Aldrich, USA) 12-well plates and culture with FibroGRO™ medium (Merck Millipore, USA), supplemented with 10 μg/mL RH-FGFb (R&D Systems, USA) and 2 μM SB431542 (STEMCELL Technologies, Saint Égrève, France). From day 13 to day 18, medium was replaced daily by fresh FibroGRO™ medium supplemented with 10 μg/mL RH-FGFb and 2 μM SB431542. From day 18 onward cells were passaged every 3–5 days using Accutase, for 5–7 min at 37°C, seeded at 5,800 cell/cm^2^ on 0.1% (w/v) gelatin-coated 6-well plates, and cultures in FibroGRO™ medium supplemented with 2 μM SB431542. SB431542 was added daily, and the medium was exchanged every 2 days. Cells were maintained under a humidified atmosphere with 5% CO_2_ at 37°C.

### Culture of primary cardiac fibroblasts

Human adult cardiac fibroblasts (aHCF) and human fetal cardiac fibroblasts (fHCF) were obtained from PromoCell (C-12375, Lot 436Z024.3) and Cell Applications (306K-05f), respectively. Cells were cultured in FibroGro LS complete media (Merck Millipore, SCMF002, USA) supplemented daily with 2 μM SB431542 (Stem Cell Technologies, 72234), with media exchange every 2 days. Cells were passaged using accutase, seeded at 5,800 cell/cm^2^ on 0.1% gelatin-coated surfaces, and maintained at 37°C under humidified atmosphere with 5% CO_2_.

### Activation assays

Cardiac fibroblasts were seeded at 5,800 cell/cm^2^ on 0.1% gelatin-coated surfaces and cultured with FibroGro LS complete media supplemented daily with 2 μM SB431542, media was exchanged every 2 days. On day 4, media was replaced with FibroGro LS complete media supplemented with 10 ng/ml TGF-β1 (R&D systems, USA) for activation or 2 μM SB431542 for control samples. Cells were further cultured for 48 h. Three independent experiments (*n* = 3) were performed for each cell source. To evaluate the effect of MK-0429 on suppressing CF activation, as pan-integrin antagonist, the drug was added 1 h after TGF-β1 followed by collection of supernatant at 48 h. Pro-collagen Iα1 secretion was assessed using Human Pro-Collagen I alpha 1 DuoSet ELISA Kit (DY6220-05, R&D systems, USA), according to the manufacturer’s instructions.

### Immunofluorescence microscopy

Cells grown on coverslips were fixed in 4% paraformaldehyde (PFA) plus 4% sucrose in phosphate-buffered saline (PBS) for 15 min at room temperature and washed three times with PBS. Prior to intracellular staining, cells were blocked and permeabilized for 30 min in 0.2% fish skin gelatin (FSG) and 0.1% TritonX-100 in PBS. Primary antibodies were then incubated for 2 h diluted in 0.1% TritonX-100 and 0.125% FSG in PBS. Cells were washed three times with PBS and incubated for 1 h with secondary antibodies diluted in 0.125% FSG in PBS. For collagen I extracellular staining, cells were blocked for 30 min with 0.2% FSG in PBS, and antibodies were diluted in 0.125% FSG in PBS. Primary and secondary antibodies were used as follows: anti-α-SMA (M085129-2, DAKO), anti-Collagen I (ab34710, Abcam, United Kingdom), AlexaFluor 488 goat anti-mouse IgG (A11001, Life Technologies, USA). Cell nuclei were counterstained with DAPI. Coverslips were mounted in ProLong Gold antifade reagent (P36934, Invitrogen, USA). Images were acquired on a Leica DMI6000B inverted microscope equipped with a Leica DFC360 FX camera, using a 20x HCX PL FLUOTAR objective, controlled with the Leica Application Suite X (LAS X) software. Images were processed using FIJI software (22) and only linear manipulations were performed.

### qRT-PCR

For samples that were not used for transcriptomic analysis, RNA was isolated using the High Pure RNA Isolation Kit (Roche, Switzerland) and quantified in NanoDrop 2000c (Thermo Scientific, USA). cDNA was synthesized from 250 ng of RNA using the Transcriptor High Fidelity cDNA Synthesis Kit (Roche, Switzerland). Polyadenylated RNAs were analyzed using the following Taqman assays: *ACTA2* (Hs00426835_g1), *COL1A1* (Hs001640 04_m1), *GATA4* (Hs00171403_m1), *ISL1* (Hs00158126_m1), *MESP1* (Hs00251489_m1), *Nanog* (Hs02387400_g1), *Nkx2.5* (Hs00231763_m1), *POSTN* (Hs01566750_m1), *POU5F1* (Hs0 0999632_g1), *TNNT2* (Hs00165960_m1), *WT1* (Hs0110375 1_m1), *RPLP0* (Hs99999902_m1), and *GADPH* (Hs9999990 5_m1) were used as control housekeeping genes. RT-qPCR reactions were performed on the LightCycler 480 Instrument II (Roche, Switzerland) and the relative gene expression was calculated using the 2^ΔΔCt^ method (23).

### Next-generation RNA sequencing

Cells were harvested using accutase (Stem cell technologies) and sedimented by centrifugation at 300 x*g*, 5 min. The supernatant was discarded, and the resulting cell pellet was washed with PBS, followed by centrifugation at 300 x*g*, 5 min. The supernatant was discarded, pellets were subjected to snap freezing and kept at −80°C. Total RNA was extracted using the RNeasy Mini Kit (Qiagen, Germany), according to the manufacturer’s instructions, and quantified using a NanoDrop 2000c (Thermo Scientific, USA). RNA quality was assessed using the Fragment Analyzer (Agilent, USA). All samples passed quality control standards of minimum concentration and RNA Quality Indicator (RQN) >9, with discrete 18S and 28S bands. cDNA libraries were generated from 500 ng of total RNA using the QuantSeq 3’ mRNA-Seq Library Prep FWD kit for Illumina (Lexogen, Austria) following the manufacturer’s instructions. Sequencing was performed on the NextSeq 500 sequencing system (Illumina, USA). Quality assessment of raw sequences and trimming were performed on the Lexogen’s QuantSeq data analysis pipeline—Bluebee. Sequences were mapped to the Ensembl Human Genome Assembly GRCh38 using the Bluebee platform^1^.

### Transcriptome dataset analysis

Transcriptome datasets were analyzed in RStudio (version 4.0.4) using Bioconductor tools. Read counts were normalized using the DESeq2 package (version 1.34.0) (24) and annotated with AnnotationDbi package (version 1.56.2) (25). Differential expression analyses were performed using DESeq2 by contrasting TGF-β-treated samples with control samples for each CF source individually. Genes with a *p*-value < 0.1 and fold-change higher than 2.0 or lower than −2.0 were considered differentially expressed. Volcano plots were generated using VolcaNoseR app (26). All identified transcripts were annotated for their cell location association using Ingenuity Pathway Analysis (IPA) (Ingenuity Systems, Qiagen, Germany). Differentially expressed genes were further analyzed using the Database for Annotation, Visualization, and Integrated Discovery (DAVID) for their association with biological processes (GO-BP) and cell component (GO-CC) gene ontology terms (27). Bubble plots were generated using the packages ggplot2 (version 3.3.5) and reshape2 (version 1.4.4). Upstream regulators’ comparative analysis was performed using IPA filtering for an overlap *p*-value > 1 (log10) and upstream regulator activation z-score > 3 as activated and those with an upstream regulator activation z-score < −3 as inhibited. Heatmaps were generated using ComplexHeatmaps package (version 2.10.0) from Bioconductor (28).

### Cardiac fibroblasts activation score calculation

For determining the CF activation score, we have considered a panel of cardiac fibroblast activation markers (*ACTA2*, *COL1A1*, *COL3A1*, *COL4A2*, *COL5A1*, *CTGF*, *FAP*, *FN1*, *FZD2*, *IL11*, *ITGA1*, *ITGA4*, *ITGA5*, *ITGB3*, *ITGB5*, *MMP2*, *P4HTM*, *PDGFA*, *PXN*, *SERPINE1*, *SPARC*, *SPP1*, *TIMP1*, *TIMP2*, *TNC*, *TNS1*, *VCAN*, and *VIM*), that were previously described to be associated with cardiac fibroblast activation (18, 20). The calculation of the CF activation scores was performed as previously reported by Peyser et al. (29). Briefly, read counts were converted to logarithmic scale, followed by z-score normalization. For each sample, z-score values correspondent to each gene described above were summed to yield the CF activation score of each sample.

### Sample preparation for mass spectrometry analysis

Cells were harvested using Versene solution (Gibco) and gentle mechanical dislodgment using a cell scraper. Cells were then sedimented by centrifugation at 300 x*g*, 5 min. The supernatant was discarded, and the resulting cell pellet was washed with PBS, followed by centrifugation at 300 x*g*, 5 min. The supernatant was discarded, pellets were subjected to snap freezing and kept at −80°C. Cell pellets were lysed in Triton X-100 lysis buffer [50 mM Tris, 5 mM EDTA, 150 mM NaCl, 1% Triton X-100 (all from Sigma-Aldrich, USA), and 1x complete protease inhibitors cocktail (Roche, Switzerland)], for 45 min at 4°C. Protein quantification was performed using Micro BCA™ Protein Assay Kit (Thermo Fisher Scientific, USA) following the manufacturer’s instructions. Proteins were precipitated using methanol, as previously described (30). Briefly, proteins were precipitated in fourfold excess of methanol, centrifuged at 9,000 x*g* for 10 s, and followed by the addition of two parts of chloroform with subsequent centrifugation. For phase separation, three parts of deionized water were added to the samples, homogenized by vigorous vortex, and centrifuged at 9,000 x*g* for 1 min. The upper phase was discarded, and three parts of methanol were added. Samples were mixed and centrifuged at 9,000 x*g* for 2 min to pellet precipitated protein. The supernatant was removed, and precipitates were dried by heating at 60°C with lids slightly ajar. For surfactant-assisted in-solution protein digestion, precipitated proteins were solubilized in 0.1% of RapiGest SF Surfactant (Waters, USA). Before digestion, samples were reduced with 5 mM of dithiothreitol (DTT) for 30 min at 60°C, alkylated with 15 mM iodoacetamide (IAA) for 30 min in dark, and boiled at 100°C for 5 min. After cooling to room temperature, protein digestion was performed by overnight incubation with trypsin (Promega; 1.2 μg per 100 μg protein) at 37°C. Trypsin inactivation was achieved by acidification with trifluoroacetic acid (TFA) at 0.5% and incubation at 37°C for 45 min. Samples were centrifuged at 16,000 x*g* for 10 min, supernatants were collected into new tubes and dried using the SpeedVac Vacufuge Plus (Eppendorf, Germany). To perform peptide cleanup samples were resuspended in 5% Formic acid (Optima LC/MS grade, Fisher Scientific, USA) using C18 microcolumns (OMIX C18 pipette tips, Agilent, USA), and then dried.

### Spectral library generation by information-dependent acquisition

A total of 5 μg from every experimental sample (total 18 samples) was used for Nano-liquid chromatography-tandem mass spectrometry (nanoLC-MS/MS) analysis on an ekspert™ NanoLC 425 cHiPLC system coupled with a TripleTOF 6,600 with a NanoSpray III source (Sciex, Framingham, MA, USA). Samples from the same condition (same cell source and same treatment) were pooled (*n* = 3). Peptides were sprayed into the MS through an uncoated fused-silica PicoTip™ emitter (360 μm O.D., 20 μm I.D., 10 ± 1.0 μm tip I.D., New Objective, Oullins, France). The source parameters were set as follows: 15 GS1, 0 GS2, 30 CUR, 2.5 keV ISVF, and 100°C IHT. A reversed-phase nanoLC-MS/MS with a trap and elution configuration, using a Nano cHiPLC Trap column (Eksigent, USA, 350 μm × 0.5 mm, ChromXP C18-CL, 3 μm, 120°Å) and NanoLC column (Eksigent, USA, 75 μm × 15 cm, ChromXP 3C18-CL-120, 3 μm, 120°Å) was performed. Water with 0.1% (v/v) formic acid (solvent A) and 0.1% formic acid in acetonitrile (solvent B) were used. Trapping was performed at 2 μL/min for 10 min using 100% (v/v) solvent A. The separation was performed at 300 nL/min applying a gradient (v/v) of solvent B as follows: 0–1 min, 5%; 1–91 min, 5–30%; 91–93 min, 30–80%; 93–108 min, 80%; 108–110 min, 80–5%; and 110–127 min, 5%. Each sample pool was subjected to two IDA runs. The mass spectrometer was set for IDA scanning full spectra (400–2,000 m/z) for 250 ms (accumulation time). The top 50 most intense precursors were selected for subsequent MS/MS scans of 150–1,800 m/z, in high sensitivity mode, for 40 ms, using a total cycle time of 2.3 s. The selection criteria for parent ions included a charge state between +2 and +5 and counts above a minimum threshold of 125 counts per second Ions were excluded from further MS/MS analysis for 12 s. Fragmentation was performed using rolling collision energy with a collision energy spread of five. The spectral library was created by combining all IDA raw files using ProteinPilot™ software (v5.0 ABSciex) with the Paragon algorithm and with the following search parameters: *Homo sapiens* from Uniprot/SwissProt database (20,394 entries, accessed on 05/01/2021); trypsin digestion; iodoacetamide cysteine alkylation; TripleTOF 6,600 equipment; and biological modifications as ID focus. After a false discovery rate (FDR) analysis, only FDR < 1% were considered (4,659 proteins). The output of these searches was used as the reference spectral library.

### Protein quantification by sequential window acquisition of all theoretical fragment ion spectra-mass spectrometry

For quantitative analysis, 5 μg of each sample were analyzed in triplicate by sequential window acquisition of all theoretical fragment ion spectra (SWATH)-MS, using the instrument setup described for the IDA runs. The mass spectrometer was operated in a cyclic data independent acquisition (DIA) similarly to the previously established method (31). SWATH-MS data were acquired with SWATH acquisition method, using a set of 64 overlapping variable SWATH windows covering the precursor mass range of 400–2,000 m/z. The variable SWATH windows were calculated using the SWATH Variable Window Calculator V1.0 (Sciex, Framingham, MA, USA) based on a reference sample. A 50 ms survey scan (400–1,800 m/z) was acquired at the beginning of each cycle, and the subsequent SWATH windows were collected from 400 to 1,600 m/z for 50 ms, resulting in a cycle time of 3.30 s. Rolling collision energy with a collision energy spread of five was used. The spectral alignment and targeted data extraction of DIA samples were performed using PeakView v.2.2 (Sciex, Framingham, MA, USA), with the spectral library as reference. For data extraction the following parameters were used: Six peptides/protein, six transitions/peptide, peptide confidence level of >96%, FDR threshold of 1%, excluding shared peptides, and extracted ion chromatogram (XIC) window of 6 min and width set at 20 ppm. Data were directly exported to Markerview 1.3.1 (Sciex, Framingham, MA, USA) and normalized using total area sums to obtain the final quantification values. A total of 4,459 proteins were quantified under these conditions.

### Proteome dataset analysis

Protein differential expression analysis was performed using the R package “DEP” (version 1.14.0) (32). Pairwise comparisons for TGF-β-treated vs. Control samples were performed for each CF source at thresholds of FDR < 0.1 and fold-change >1.5. Pearson correlation coefficients were calculated in Perseus software environment, using log-transformed protein intensities (33). Proteins were annotated for their cell location using Ingenuity Pathway Analysis (IPA). The association of upregulated differential expressed proteins (DEPs) with fibrotic conditions was performed using the DisGeNET database, by filtering for association with pulmonary fibrosis, cardiac fibrosis, hepatic fibrosis, or renal fibrosis (34). The availability of drugs to target the upregulated DEPs was assessed using IPA and a drug-gene interaction database the Open Targets Platform (35–37).^2^ The co-expression network of upregulated proteins was generated using STRING (Search Tool for the Retrieval of Interacting) (version 11.5), setting the minimum required interaction score for high confidence (0.7), and hiding disconnected nodes (38–48). Volcano plots were generated using VolcaNoseR app and heatmaps were generated using ComplexHeatmaps package (version 2.10.0) from Bioconductor (26, 28).

## Results

### Transcriptomic and proteomic analysis of activated and quiescent cardiac fibroblasts

**FIGURE 1:**
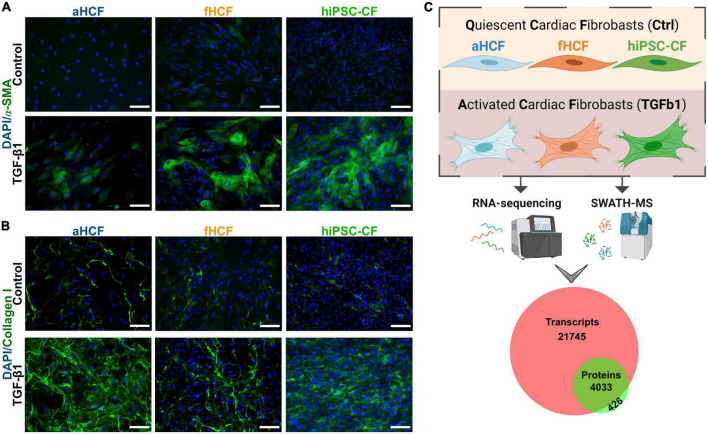
Transcriptomic and proteomic analysis of activated and quiescent CF **(A)** TGF-β1 treated CF show hallmarks of CF activation: Formation of smooth muscle actin (α-SMA) stress fibers and **(B)** increased collagen I deposition. Scale: 100 μm. **(C)** Overview of study design: three sources of CF were used for a comparative analysis of the transcriptome (through high-throughput RNA-sequencing) and the proteome (through SWATH-MS) of a quiescent state (non-activated) and an activated state. Three independent experiments were performed for each CF source. The analysis resulted in a dataset of 25,779 transcripts and 4,659 proteins. Venn diagram shows coverage and overlap between total identified transcripts and proteins.

Three cell sources were used to investigate the molecular remodeling occurring upon cardiac fibroblast activation. We used human primary cells from adult (aHCF) and fetal (fHCF) origin, and hiPSC-CF. Quiescent hiPSC-CF were generated by differentiation of hiPSC into CF *via* temporal modulation of Wnt/β-catenin signaling, based on previous reports (16, 21) (Supplementary Figure 1). The three CF sources were maintained under the same culture conditions, including low-serum medium, low percentage of gelatin coating, and daily supplementation with a low concentration of a TGF-β1 inhibitor. These conditions were shown to be essential for the maintenance of the quiescent CF phenotype *in vitro* (Figures 1A,B). Cell activation was induced by treatment with TGF-β1, which resulted in formation of α-smooth muscle actin (α-SMA) stress fibers and increased collagen I deposition, for all three cell sources (Figures 1A,B). The induction of these hallmarks of CF activation was also confirmed at the gene expression level, with a significant upregulation of *ACTA2* and *COL1A1* upon treatment, except for aHCF, where despite a similar trend, a more modest and non-statistically significant fold-change was observed for both genes (Supplementary Figures 2A,B). A comprehensive study of the molecular remodeling upon CF activation was performed at both transcriptomic (high-throughput RNA sequencing) and proteomic (SWATH-MS) levels, analyzing samples of quiescent CF (control) and TGF-β1 treated CF (Figure 1C). Pairwise comparisons of biological replicates revealed strong correlations between samples, as demonstrated by the Pearson correlation coefficients above 0.9 and 0.7, for transcriptome and proteome, respectively (Supplementary Figure 3). The analysis led to the identification and quantification of 25,779 transcripts and 4,659 proteins. For 91% of the identified proteins, it was possible to quantify the respective transcript abundance (Figure 1C).

### Different cardiac fibroblasts sources present distinct levels of quiescence under *in vitro* culture conditions

**FIGURE 2:**
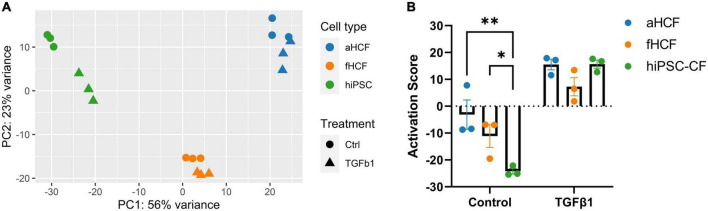
Transcriptome dataset reveals distinct CF quiescence levels in control samples **(A)** Principal component analysis of normalized read counts for all samples. **(B)** Read counts for genes previously associated with CF activation were converted into z-scores. For each CF source, all z-scores were summed to yield the CF activation score. Two-way ANOVA followed by Tukey’s multiple comparisons test reveals significant differences in quiescent state for control samples, *p-value < 0.05; **p-value < 0.01. Panel (A) was created in Biorender (copyright agreement number: SU24OC0BPY).

The obtained transcriptome dataset was assessed through principal component analysis (PCA) of all genes, where samples clustered primarily according to their cell source (Figure 2A). Within each cell source, it was possible to discriminate samples that were TGF-β-treated from the respective control samples. The largest distance observed between control and treated groups was for hiPSC-CF, suggesting a stronger modulation upon TGF-β exposure, relative to CF from primary origin. For fHCF and aHCF, control and treated samples also clustered independently, except for one sample from each group of aHCF that was overlapping. Thus, even applying consistent culture conditions and procedures, primary cells may acquire a less quiescent state when maintained *in vitro*. To assess if that was the case, we determined a CF activation score for each sample, based on the expression levels of a panel of genes previously associated with CF activation (Supplementary Figure 4A) (18, 20). This analysis revealed that all CF sources presented a significant increase in the CF activation score upon treatment. Also, the values obtained for the control samples allowed a comparison regarding the quiescent state of these cells. Here, hiPSC-CF displayed a consistent and significantly lower CF activation score relative to aHCF and fHCF (Figure 2B). Furthermore, under the same culture conditions hiPSC-CF showed a higher population doubling level and a shorter doubling time in comparison to primary cell sources (Supplementary Figure 1).

### Transcriptome remodeling reveals a convergent response of cardiac fibroblasts sources upon activation

**FIGURE 3:**
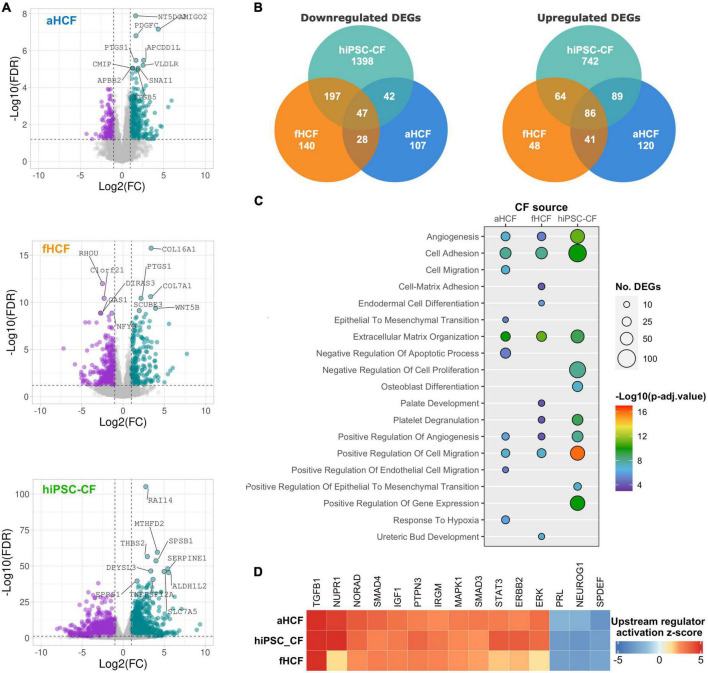
Transcriptional changes upon CF activation reveal a convergent remodeling in different CF sources **(A)** Volcano plots of differentially expressed genes between control and TGF-β-treated conditions, for each CF source. Downregulated genes are in purple and upregulated genes in green, non-significant changes are in gray. The top 10 modulated genes are annotated. Applied thresholds for fold-change: −1 and 1; significance: 1.2. **(B)** Venn diagrams showing the comparison of upregulated DEGs and downregulated DEGs between the CF sources. **(C)** Bubble plot shows the top 10 biological processes enriched for each CF source. Bubble size indicates the number of genes associated with each term and bubble color indicates the significance of the enrichment. **(D)** Heatmap from a comparative analysis of upstream regulators activation z-score of top 15 upstream regulators determined by ingenuity pathway analysis.

To understand differences and similarities in response between the CF sources to TGF-β treatment, we analyzed each CF transcriptome dataset independently (Figure 3A). Differential gene expression analysis revealed 2,664 differentially expressed genes (DEGs) for hiPSC-CF, among which 1,683 were downregulated and 981 upregulated. It was clear that hiPSC-CF showed the strongest response to TGF-β treatment, presenting higher fold-changes and higher statistical significance values, relative to the primary cells. Indeed, aHCF displayed the lower level of modulation upon TGF-β treatment, with 560 DEGs identified, among which 224 were downregulated and 336 upregulated. For fHCF, 651 DEGs were identified, 412 were downregulated, and 239 were upregulated. The three CF sources shared 86 upregulated DEGs and 47 downregulated DEGs (Figure 3B).

To compare the response to TGF-β between the different CF sources, a functional enrichment analysis based on gene ontology (GO) annotation was performed using the complete set of DEGs identified for each CF source. Considering the top 10 most significantly enriched biological processes (GO-BP) from each dataset, we found that all cell sources presented an enrichment for processes related to angiogenesis, cell adhesion, ECM organization, and cell migration (Figure 3C). Accordingly, cell component (GO-CC) enrichment analysis revealed significant modulation of genes associated with the plasma membrane, ECM, and the extracellular space in all CF sources (Supplementary Figure 5B). Considering only the top 10 enriched GO-BP for all CF sources, hiPSC-CF and aHCF also shared enrichment for endothelial-to-mesenchymal transition, and hiPSC-CF and fHCF shared enrichment for platelet degranulation processes.

We used the Ingenuity Pathway Analysis (IPA) to predict the main upstream regulators activated and inhibited in response to TGF-β for the three CF sources (Figure 3D). The top 15 upstream regulators that were activated in all CF sources included transforming growth factor beta 1 (TGFB1), nuclear protein 1 (NUPR1), non-coding RNA activated by DNA damage (NORAD), the protein tyrosine phosphatase non-receptor type 3 (PTPN3), the SMAD family member 4 (SMAD4), the signal transducer and activator of transcription 3 (STAT3), the insulin-Like Growth Factor 1 (IGF1), the Erb-B2 receptor tyrosine kinase 2 (ERBB2), the immunity-related GTPase M (IRGM), extracellular signal-regulated kinase (ERK), the mitogen-activated protein kinase 1 (MAPK1), and the SMAD family member 3 (SMAD3). By contrast, the top upstream regulators predicted to be inhibited in all CF included, neurogenin 1 (NEUROG1), the SAM pointed domain containing ETS transcription factor (SPDEF), and prolactin (PRL).

Overall, this comparative analysis revealed a consistent and convergent gene expression response to TGF-β between all CFs sources, both in terms of the main biological processes being modulated and the key regulators driving such phenotypic changes.

### Extracellular matrix and plasma membrane remodeling is a signature of cardiac fibroblasts activation

**FIGURE 4:**
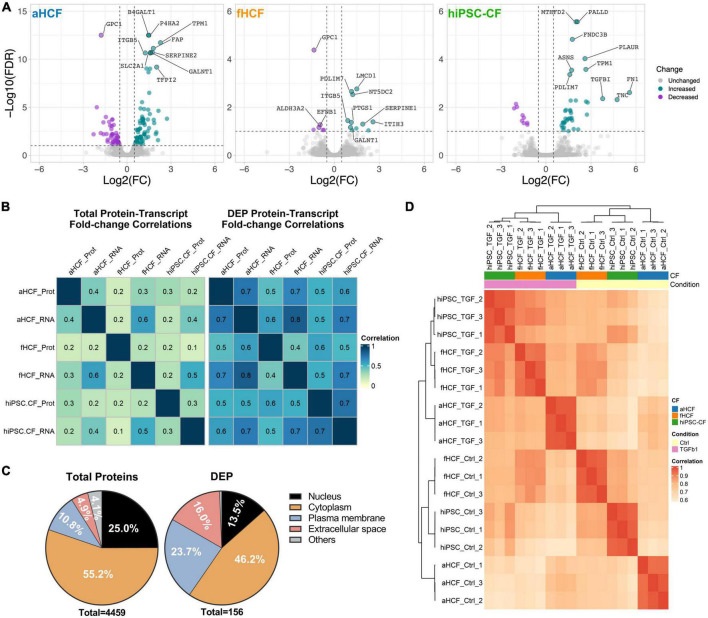
Proteome remodeling upon CF activation **(A)** Volcano plots of differentially expressed proteins between control and TGF-β-treated samples, for each CF source. Downregulated proteins are in purple and upregulated proteins in green, non-significant changes are in gray. The top 10 modulated proteins are annotated. Applied thresholds for fold-change: −0.5 and 0.5; significance: 1. **(B)** Heatmaps show the Pearson correlation coefficient of fold-changes between transcriptome and proteome considering total protein-transcript pairs (*n* = 2,952) on the left panel; and considering protein-transcript pairs corresponding to DEPs (*n* = 97) on the right panel. **(C)** Representativity of cellular locations within the total quantified proteins dataset and within DEPs dataset. **(D)** Hierarchical clustering of Pearson correlation coefficient considering DEPs annotated as ECM-proteins or membrane proteins.

We applied a quantitative untargeted proteomic analysis (SWATH)-MS to characterize the proteome remodeling upon CF activation to identify potential targets to modulate the CF-active state. When comparing the quiescent state CF and activated state CF, we identified 157 differentially expressed proteins (DEPs), 123 on the aHCF dataset, 17 for fHCF, and 46 for hiPSC-CFs (FDR < 10%) (Figure 4A). The serpin family E member 1 (Serpine1), integrin subunit beta 5 (ITGB5), and the 5’-Nucleotidase Domain Containing 2 (NT5DC2) were upregulated in all CF (Supplementary Figure 6A).

The correlation values between the changes in transcriptome and in proteome, upon treatment, increased considerably when only the DEPs were considered (Figure 4B and Supplementary Figure 6B). Particularly, the Pearson correlation coefficient (PCC) for the aHCF and hiPSC-CF datasets increased from 0.3 to 0.7. More modest correlation values were observed between the fHCF datasets, with an increase from 0.224 to 0.433.

When considering the complete list of DEPs, hierarchical clustering of the different samples revealed that the main separation between groups was driven by cell source (Supplementary Figure 6C). This observation was also in line with what was previously described for the transcriptome data (Figure 2A). At the transcriptomic level, we have observed enrichment for processes associated with two cell compartments: extracellular space and plasma membrane. The prevalence of proteins associated with ECM and plasma membrane increased from 15 to 40% when considering the DEPs dataset, similar to the transcriptome dataset, further suggesting the relevance of such components in the response to treatment (Figure 4D and Supplementary Figure 6D). Also, filtering the list of DEPs for proteins annotated as part of extracellular space and plasma membrane and calculating PCC between samples, enabled the segregation of quiescent and TGF- β-treated samples (Figure 4C). This was especially relevant for hiPSC-CF and fHCF which clustered together for both control and TGF-β1 treated samples. aHCF samples group together independently of treatment. Taken together, these observations suggest that TGF-β treatment, leads to significant remodeling of the plasma membrane and ECM proteins, by inducing the CF activation pathways, where once again convergent responses were observed for the three different CF sources evaluated. These are usually highly tractable proteins, which can potentially represent relevant targets to be explored in a therapeutic context.

### Target prioritization for cardiac fibroblasts state modulation in cardiac fibrosis

**FIGURE 5:**
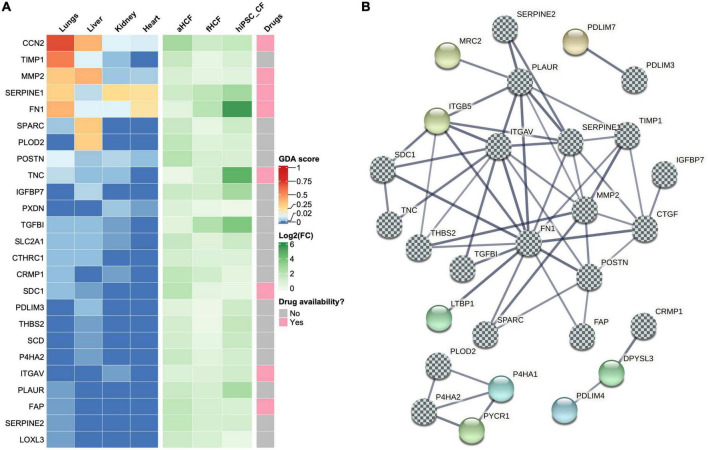
Identification of potential therapeutic targets in cardiac fibrosis **(A)** Heatmaps show upregulated DEPs associated with fibrotic conditions. In left panel each column represents a gene-disease association score (GDA score) attributed by DisGeNET. Center panel shows the log2 (fold-change) for each CF source. Right panel shows drug availability for direct targeting of those proteins. **(B)** Co-expression network of upregulated proteins associated with fibrotic conditions. The chess pattern highlights fibrosis-associated proteins.

We then interrogated the set of upregulated DEPs for their previous association with fibrotic conditions, using a gene-disease association database DisgesNET (34). DisGeNET is one of the largest publicly available collections of genes associated with human disease from curated sources and scientific literature. Of the 105 upregulated DEPs, 25 proteins were already linked with fibrotic conditions, including cardiac fibrosis (Figure 5A). For example, fibronectin 1 (FN1), plasminogen activator inhibitor type I (SERPINE1), and the cellular communication network factor 2 (CCN2, also known as CTGF) were already associated with fibrosis in the heart. Other proteins, such as tissue inhibitor metallopeptidase 1 (TIMP1), matrix metallopeptidase 2 (MMP2), pro-collagen-lysine-2-oxoglutarate 5-dioxygenase 2 (PLOD2), secreted protein acidic and cysteine rich (SPARC), Tenascin C (TNC), insulin-like growth factor binding protein 7 (IGFBP7), and periostin (POSTN) were associated with other fibrotic conditions but not in the cardiac context, according to this database. Proteins such as peroxidasin (PXDN), syndecan 1 (SDC1), the collagen triple helix repeat containing 1 (CTHRC1), solute carrier family 2 member 1 (SLC2A1), the transforming growth factor beta-induced protein (TGFBI), integrin subunit alpha V (ITGAV), fibroblast activation protein alpha (FAP), lysyl oxidase like 3 (LOXL3), urokinase plasminogen activator surface receptor (PLAUR/uPAR), glia-derived nexin (SERPINE2), collapsin response mediator protein 1 (CRMP1), PDZ and LIM domain 3 (PDLIM3), prolyl 4-hydroxylase subunit alpha 2 (P4HA2), thrombospondin 2 (THBS2), and stearoyl-CoA desaturase (SCD), presented also previous associations to all fibrotic conditions although with lower score values.

The association of some DEPs with several fibrotic conditions further highlights the relevance of the generated datasets for the identification of putative targets in cardiac fibrosis. To expand this panel, we investigated which other proteins in our DEPs dataset presented direct protein-protein interactions to the ones identified as associated with fibrosis (Figure 5B and Supplementary Figure 7). STRING analysis showed an interconnection of a large group of proteins (19 in total), from those three proteins were not previously linked to fibrotic conditions: (1) mannose receptor C type 2 (MRC2) that acts also as a collagen receptor was identified as an interacting partner of PLAUR; (2) latent transforming growth factor beta binding protein 1 (LTBP1), which is a key regulator of TGF-β1, was identified as an interacting partner of FN1; and (3) integrin subunit beta 5 (ITGB5) was identified as an interacting partner of several proteins linked to fibrosis, namely PLAUR, ITGAV, SERPINE1, FN1, THBS2, and SDC1. STRING also revealed the interaction between PDLIM3 and PDZ and LIM domain 7 protein (PDLIM7). CRMP1 was found to interact with dihydropyrimidinase-like3 protein (DPYSL3), a molecule that is involved in cytoskeleton remodeling, which subsequently interacts with the PDZ and LIM domain 4 (PDLIM4). PLOD2 and P4HA2, where found to interact with each other and are within the same cluster interacting with two more proteins: prolyl 4-hydroxylase subunit alpha 1 protein (P4HA1) and pyrroline-5-carboxylate reductase 1 (PYCR1). Thus, the co-expression network analysis allowed to identify eight proteins that interact with proteins previously associated with fibrosis. These proteins also represent a valid option to explore as targets in cardiac fibrosis, expanding our panel of potential targets to 33 proteins.

We then investigated how many proteins from the panel of those 33 putative targets had already drugs directly targeting them, using a gene-drug interaction database, IPA, and the open targets platform (Figure 5A). Only nine were found to have approved or under study drugs: ITGAV, CCN2, FAP, FN1, ITGB5, MMP2, SDC1, SERPINE1, and TNC. From those only two had fibrotic indications, STX-100 targeting ITGAV and Pamrevlumab targeting CCN2, both for pulmonary fibrosis indications. A feasibility study was conducted using MK-0429, a small molecule pan-integrin inhibitor (49) to validate the ITGAV/ITGB5 targets. For this, hiPSC-CF were activated as described above and cultured with or without MK-0429 (Supplementary Figure 8). The inhibitory effect of this small molecule was observed by 1.6-fold decrease in pro-collagen Iα1 secretion, relative to TGf-β1-treated cells. This observation also highlights the potential of hiPSC-CF for drug-testing studies. Nonetheless, the reduced availability of drugs targeting the identified proteins, and the identified candidates not yet explored in the cardiac context shows the urgency to discover therapeutic solutions for cardiac fibrosis. Furthermore, the panel of 33 proteins here described can be useful as novel targets for new drug discovery campaigns or drug repositioning initiatives.

## Discussion

Cardiac fibrosis is an unmet medical need affecting millions of patients worldwide. Since activated CF are one of the major cellular drivers of cardiac fibrosis development, finding novel targets to modulate CF state represents an opportunity to treat these patients. Reasons for the current shortage of actionable targets in cardiac fibrosis include the lack of sustainable tissue-specific *in vitro* models of CF and reliable cell culture systems (14–16). Here, the use of primary cells for instance as led to some controversy in the field, namely due to the challenges in defining cell identity markers (11). Many of the traditionally used CF markers (e.g., vimentin, CD90, and FSP1) have been shown to be also expressed in other cardiac cells, potentially hampering our understanding of CF role in cardiac fibrosis (11, 50). The aim of this study was to identify potential targets for cardiac fibrosis, by leveraging the use of human *in vitro* cell models from different cells sources, and thus minimizing potential pitfalls related with artifacts of a single cell source. Here, we report a comprehensive transcriptome and proteome analysis of activated CF from three distinct cell sources (aHCF, fHCF, and hiPSC-CF) cultured under the same defined conditions.

Human-induced pluripotent stem cell-derived CF were included in this study as an alternative CF source capable of overcoming some of the limitations of working with primary cells, as suggested by previous reports (14, 16, 51, 52). Transcriptomic analysis revealed a convergent response of all CF sources to TGF-β1 treatment through modulation of biological processes known to be associated with CF activation, such as cell migration, ECM remodeling, and cell adhesion (3, 8, 53). Moreover, using the transcriptomic datasets, we assessed the activation level of our CF samples by defining a CF activation score based on markers associated with CF activation. hiPSC-CF exhibited lower activation levels than primary cells under the same *in vitro* culture conditions, while keeping similar responsiveness to pro-fibrotic stimuli. These results confirm the responsiveness of epicardial-derived CF, as suggested by Floy et al. and the suitability of using these cells in anti-fibrotic screenings (52). Here, we show that the advantages of using hiPSC-CF for preclinical research are not only related to scalability but also due to the fact that they provide a wider assay window (difference between control and treated samples) in comparison to primary cells (54). Recently, hiPSC-derived CF have been used in drug screenings and in the development of heterotypic advanced cell models of cardiac fibrosis, contributing toward more physiologically relevant models and a better understanding of disease mechanisms (20, 55–59). Moreover, to our knowledge this is the first time the whole transcriptome and proteome of hiPSC-CF is compared to primary CF under activation conditions. Noteworthy, it is also the first comparison in which primary cells and hiPSC-CF are cultured under the exact same conditions.

One of the most striking features of cardiac fibrosis is the excessive accumulation of ECM proteins. Accordingly, here we found that filtering for proteins associated with extracellular space and the plasma membrane enabled the separation between the two tested conditions independently of the cell type (control vs. TGF-treated samples), revealing that even when considering the whole proteome, the most marked changes under activation happen in the matrisome (60). Indeed, the examination of protein-protein interactions defined a large group of matrisome proteins involved in ECM synthesis, degradation, interaction, and regulation, including ITGAV, ITGB5, PLAUR, TNC, SERPINE1, SERPINE2, LTBP1, SPARC, FAP, MMP2, POSTN, SDC1, CTGF, THBS2, MRC2, IGFBP7, TGFBI, and TIMP1. Most likely the interplay between these proteins contributes for the persistence of CF in the active state (61). Some of these proteins are already being explored for the treatment of fibrosis in a clinical setting. For example, a monoclonal antibody against CTGF (also known as CCN2) is currently being evaluated in phase III clinical trial for the treatment of idiopathic pulmonary fibrosis (IPF) (62, 63), which showed also an improved cardiac repair through the downregulation of profibrotic and inflammatory genes in a mouse model of myocardial infarction (MI) (64). FAP is widely explored as relevant target for fibrosis indications. Particularly in the heart, FAP-expressing fibroblasts have been found in samples from human hearts after MI, but not in healthy hearts (65, 66). Recently, promising data has been published on the development of anti-FAP CAR-T cells to treat cardiac fibrosis, demonstrating that effective elimination of FAP-positive cells from the injury site in a mouse model of heart failure improved cardiac compliance (67, 68). Interestingly, the physiological function of FAP appears to depend on its association with other molecules such as integrins and PLAUR, proteins that we have also identified as upregulated in our dataset (69, 70).

In fact, we identified four proteins belonging to the fibrinolytic system (SERPINE1, SERPINE2, PLAUR, and MRC2), suggesting dysregulation of this system upon CF activation. Both SERPINE1 and SERPINE2 can inhibit the activation of plasminogen into plasmin with consequent inhibition of ECM degradation mechanisms (71). Therefore, targeting these proteins could potentially promote the degradation of fibrotic tissue (72). On the other hand, PLAUR and MRC2 are involved in the activation of plasminogen and, although the benefit of targeting these proteins in fibrosis remains to be fully demonstrated, encouraging results have been reported for cancer settings (73–76). Also, in a mouse model of renal fibrosis, disruption of MRC2 (genetic knock-out or chemical inhibitor) has been shown to enhance the fibrotic phenotype (77).

Within the matrisome, integrins are thought to be involved in the persistence of CF in the activated state, with two main molecules being highlighted in our datasets, ITGAV and ITGB5. Pharmacological inhibition of these using cilengitide resulted in improved cardiac function in a mouse model of MI and a decrease in the expression of markers associated with CF activation *in vitro* (78, 79). In pulmonary fibrosis, the pan-integrin inhibitor MK-0429 and an antibody described by Zhang et al. team, showed promising results *in vitro*, raising the interest in assessing their potential in cardiac fibrosis models (49). Integrin-mediated activation is also stimulated by LTBP1 (80–82), which was identified to be upregulated in our dataset as well. Although no known drugs targeting LTBP1 are yet available, selective inhibition of this protein may be an interesting approach to reduce TGF-β1 activation (83) given its role as regulator of TGF-β pathways, through interactions with ECM proteins (84–87), namely collagen-interacting proteins (e.g., FN1, MMP2, and thrombospondins) (88–93). Collagen is actually the most abundant ECM protein on the cardiac fibrotic scar (94). Consistent with that, we identified several proteins associated with collagen synthesis (P4HA2, P4HA1, and PLOD2) and cross-linking (FN1, LOXL3, and PXDN) to be upregulated (95–98). Interfering with collagen synthesis and cross-linking has been proposed to be an interesting approach to modulate cardiac fibrosis (99). Valiente-Alandi et al. showed that inhibition of FN1 polymerization resulted in reduced deposition of ECM, CF proliferation and migration *in vitro*, while attenuating the fibrotic markers in animal models (100). Similar encouraging results, have also been reported for liver and kidney fibrosis (101, 102). PYCR1, a protein that indirectly fuels collagen production through proline metabolism (103–105), was also found to be upregulated and has recently been proposed as potential target for pulmonary fibrosis (106–108). Interestingly, we also observed the upregulation of another metabolic protein: the glucose transporter SLC2A1, also known as GLUT1. The highly proliferative and secretory phenotype of myofibroblast is likely to increase the energy demands, with consequent upregulation of proteins mediating carbon-sources uptake (109–111). Indeed, Andrianifahanana et al. have shown that SLC2A1 is required for the development of the profibrotic effects of TGF-β1 and reported its upregulation in patients with idiopathic pulmonary fibrosis (112). Despite the potential challenges of developing therapies targeting a glucose transporter, its inhibition in an *in vitro* model of hyperglycemia resulted in attenuated fibrosis progression (113). Additional interesting targets revealed in our datasets include CTHRC1, which has been linked to physiological and pathologic conditions (114, 115), including cancer and fibrosis (115–117). Reports of their protective effect or pathological contribution on the development of fibrosis are contradictory (118–121). However, CTHRC1-positive CF have been identified in scars of hearts of mouse and swine models of myocardial infarction (MI), as well as, in human samples from cardiovascular disease patients (122, 123). Importantly, CTHRC1 has been reported as being involved in the mechanism of action (MoA) of pirfenidone, an anti-fibrotic agent approved for treatment of idiopathic pulmonary fibrosis and an ongoing study showing promising results for heart conditions (124, 125). Other proteins identified here have somehow been linked to fibrosis or fibroblast activation, although the underlying mechanisms are not well studied. This group of proteins include CRMP1, DPYSL3, IGFBP7, P4HA1, P4HA2, PDLIM3, PDLIM4, PDLIM7, PLOD2, PXDN, PYCR1, SCD, and SDC1 (126–128).

Considering the intersection of our datasets for all CF sources, insulin growth factor protein, IGF-1, was identified in the top activated upstream regulators in all CF sources, having been also previously implicated in cardiac fibrosis by other authors (128–130). From the differentially expressed proteins lists, only three proteins were identified as upregulated in all CF sources: ITGB5, SERPINE1, and NT5DC2. Although, the relevance of targeting ITGB5 and SERPINE1 was already described above, scarce information is available on NT5DC2. NT5DC2 is a 5′-nucleotidase that can catalyze the hydrolysis of nucleotides and has been associated with psychiatric disorders and cancer (131–134). NT5DC2 was also previously detected as transcriptionally upregulated in studies of atrial fibrillation and pulmonary fibrosis, but little attention has been given to NT5DC2 as a potential target (135–137).

Collectively, here we provide transcriptomic and proteomic datasets of quiescent and activated CF that showed physiological properties under defined culture conditions. These datasets were obtained from three different human cell sources and may provide the community with a relevant tool to strengthen our understanding of human CF biology. From the proteome datasets we prioritized 33 proteins as potential targets for cardiac fibrosis. Most of these proteins are currently underexplored as therapeutic targets in the cardiac fibrosis setting but some have been explored for other fibrotic or oncologic indications, namely FAP, PLAUR, ITGB5, and MRC2. Strategies to minimize artifacts associated with CF *in vitro* culture were put in place, namely using low-serum conditions and a soft matrix substrate to minimize basal activation levels. Nevertheless, one should not disregard potential unforeseen bias typical of *in vitro* cell models, meaning that further confirmatory studies on these targets will be key to fully assess the relevance of the generated data in the context of pathological cardiac fibrosis. We believe that drugs developed for these targets under oncologic programs can be useful in cardiac fibrosis, as many of them are targeting cancer associated fibroblasts which display characteristics of myofibroblasts (138, 139). While our data and previous reports demonstrate the complexity of the fibrotic response, including positive and negative feedbacks, we propose the exploitation of the prioritized targets as part of drug repositioning strategies or novel drug discovery campaigns, to open new avenues toward effective anti-fibrotic therapeutics.

## Data availability statement

The datasets presented in this study can be found in online repositories. The names of the repository/repositories and accession number(s) can be found below: https://www.ncbi.nlm.nih.gov/geo/, GSE202714 and https://www.ebi.ac.uk/pride/archive/, PXD034244.

## Author contributions

DS, MMS, and MRM: conceptualization and writing—original draft. DS, MRM, MMS, and MS: methodology. MRM and CD: experimental work. DS and MRM: formal analysis and visualization. MRM, DS, and MMS: writing–original draft. DS, MMS, CD, AB, RH, and MS: writing—review and editing. DS, AB, and MS: funding acquisition. AB, DS, RH, MMS, and MS: supervision. All authors contributed to the article and approved the submitted version.

## Footnotes

1
https://lexogen.bluebee.com/

2
https://www.dgidb.org
