# Supplementary material for: Transcriptome and proteome profiling of activated cardiac fibroblasts supports target prioritization in cardiac fibrosis
Source: Front Cardiovasc Med. 2022 Dec 1;9:1015473. doi: 10.3389/fcvm.2022.1015473 (PMC9751336; doi:10.3389/fcvm.2022.1015473)
Supplement: Supplementary file 1 [file Data_Sheet_1.docx]

Supplementary Figures Captions

**Figure S1.** Characterization of CF cell sources **(A)** Schematic depiction of differentiation stages and respective expected gene expression changes along the differentiation process. **(B)** Representative bright-field images showing stage-specific morphological changes during differentiation (scale: 200 µm). **(C)** Stage-specific gene expression levels along the differentiation: pluripotency (*Nanog* and *POU5F1*), cardiac progenitors (*MESP1*, *ISL1* and *Nkx2.5*), epicardial (*GATA4*, *WT1* and *TNNT2*), and cardiac fibroblasts (*POSTN*). Heatmap of mean relative expression assessed by quantitative real-time PCR (n=3). **(D)** Representative bright-field images showing morphology of primary cells under *in vitro* culture conditions (scale: 200 µm). **(E)** Cumulative population doubling levels (PDL) plotted to the number of passages in subculture for each CF source. Data collected during cell expansion and maintenance. **(F)** Mean of population doubling time in hours for each CF source.

**Figure S2.** Expression of genes associated to activated CF phenotype **(A)** *ACTA2* **(B)** *COL1A1*. Data are presented as mean ± SEM and p-values from Bonferroni’s post hoc multiple comparisons test after two-way ANOVA (**p < 0.01, ***p < 0.001, ****p<0.0001, ns = no significant difference between Control and TGFβ1 samples).

**Figure S3.** Heatmaps show the Pearson correlation coefficient between samples for **(A)** transcripts dataset and **(B)** proteins dataset.

**Figure S4.** Heatmap shows the average z-score for each marker used on CF activation score (A) at the transcriptome level (B) and at the proteome level.

**Figure S5.** Gene ontology analysis (GO) enrichment analysis for cell component. Bubble plot presents the top 10 enriched cell components for each CF source. Bubble size indicates the number of transcripts that are associated with each term and bubble color indicates the –log_10_(p-adjusted-value) of the enrichment.

**Figure S6.** **(A)** Comparison of upregulated DEPs between the three CF sources **(B)** Comparison of fold-changes between transcriptome (x-axis) and proteome (y-axis) for each CF source and Pearson correlation coefficients considering all identified proteins on the left panel and the total DEPs on the right panel. DEPs for each CF source are highlighted in color. **(C)** Hierarchical clustering of Pearson correlation coefficient considering total DEPs. **(D)** Representativity of cellular localizations within the total quantified transcripts dataset and within DEG dataset.

**Figure S7.** Co-expression network considering all upregulated proteins. The chess pattern highlights fibrosis-associated proteins.

**Figure S8**. Quantification of pro-collagen Ia1 in cell culture supernatants of hiPSC-CF cultured in different conditions: (i) non-treated; (ii) treated with TGF-β1; (iii) treated with TGF-β1 and SB43154; (iv) treated with TGF-β1 and MK-0429. Two independent experiments were performed. Data are presented as mean ± SD.
