# Supplementary figures and images for: Transcriptome and proteome profiling of activated cardiac fibroblasts supports target prioritization in cardiac fibrosis
Source: Front Cardiovasc Med. 2022 Dec 1;9:1015473. doi: 10.3389/fcvm.2022.1015473 (PMC9751336; doi:10.3389/fcvm.2022.1015473)

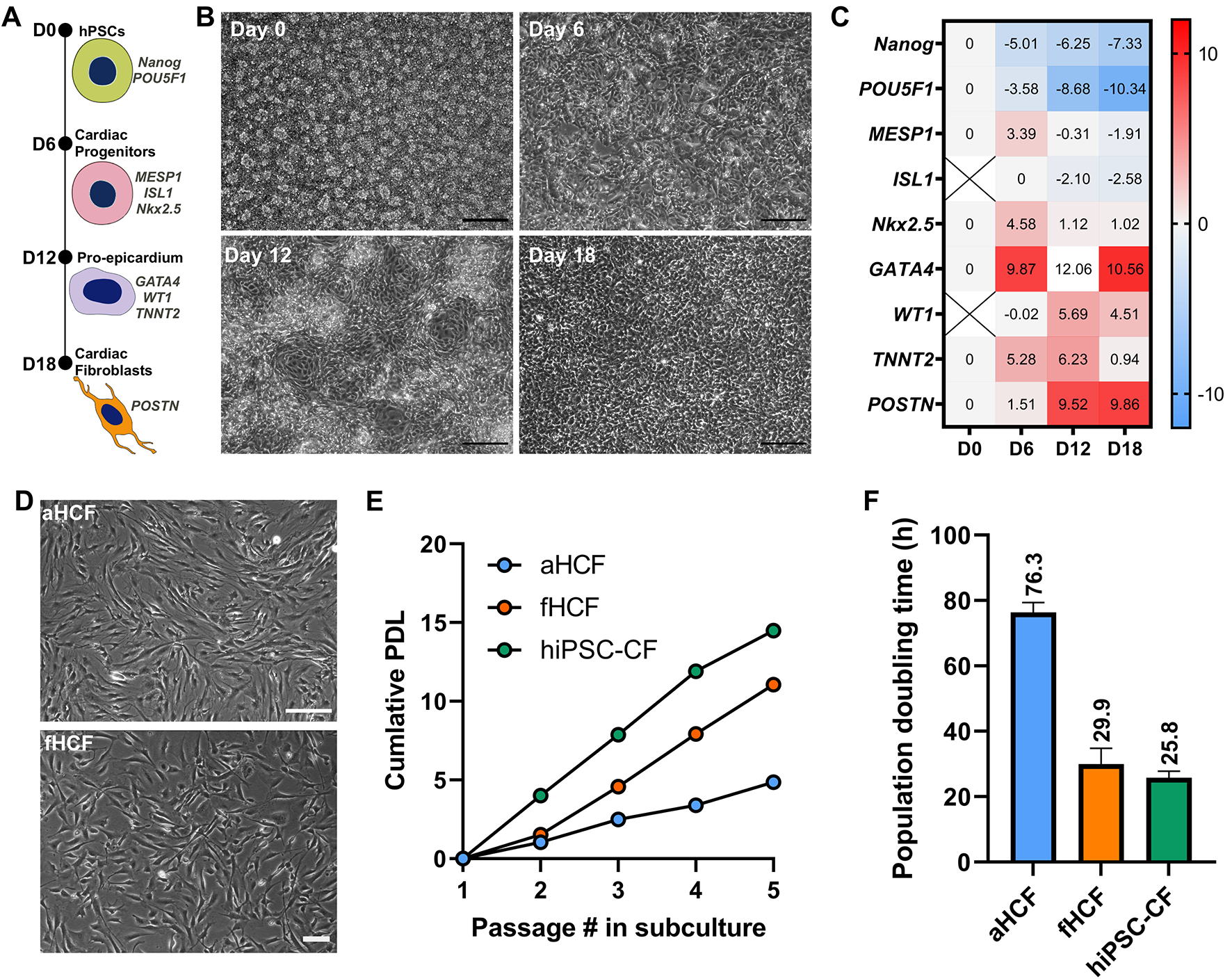

Supplement: Supplementary file 2 [file Image_1.TIFF]

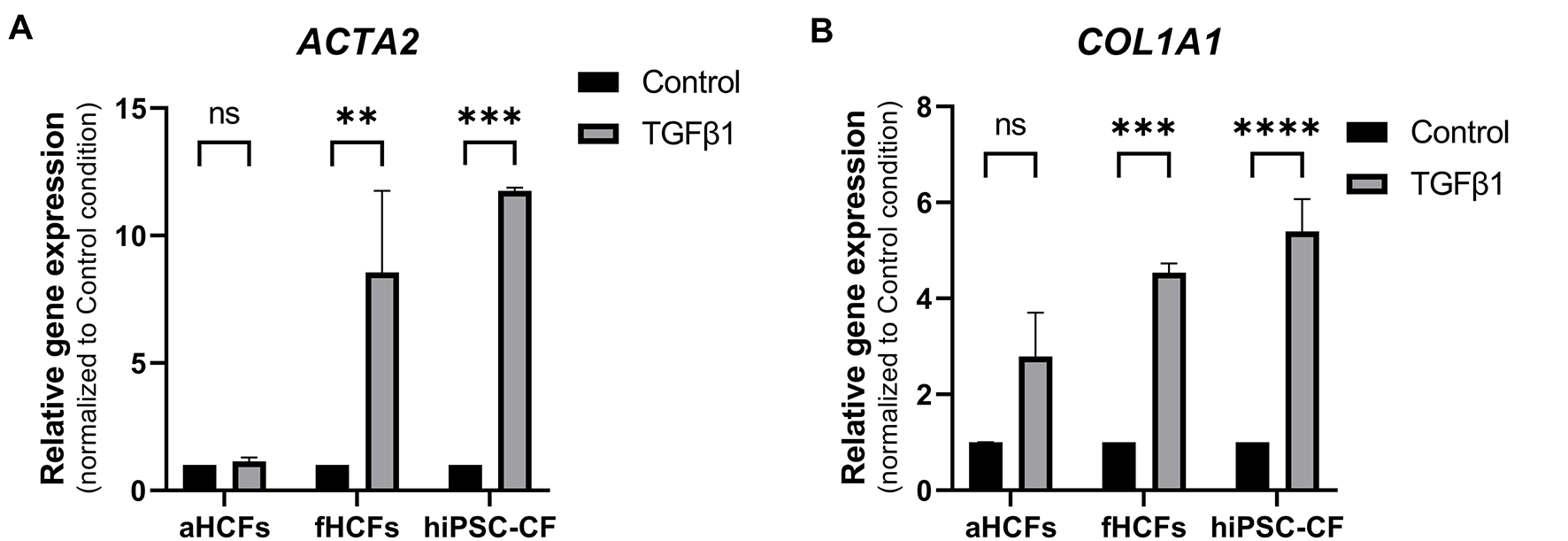

Supplement: Supplementary file 3 [file Image_2.TIFF]

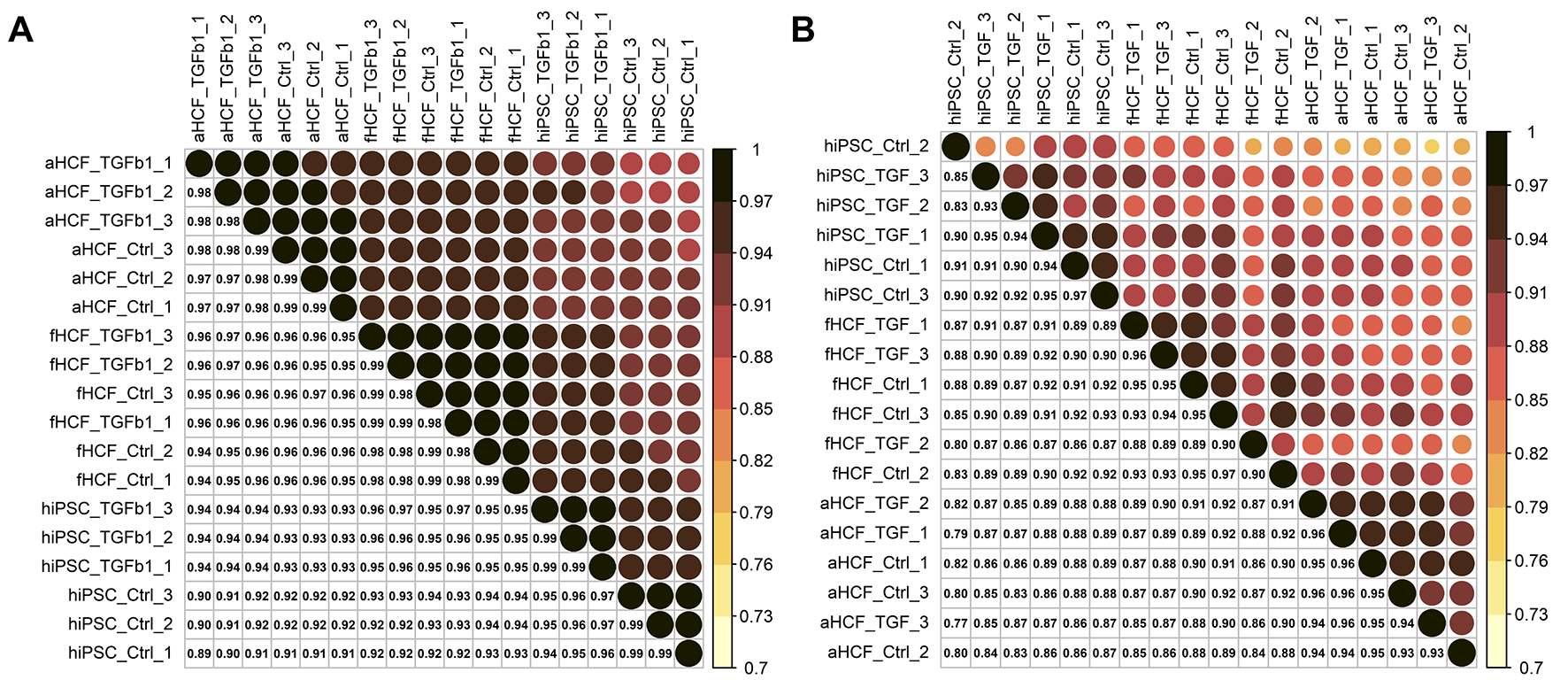

Supplement: Supplementary file 4 [file Image_3.TIFF]

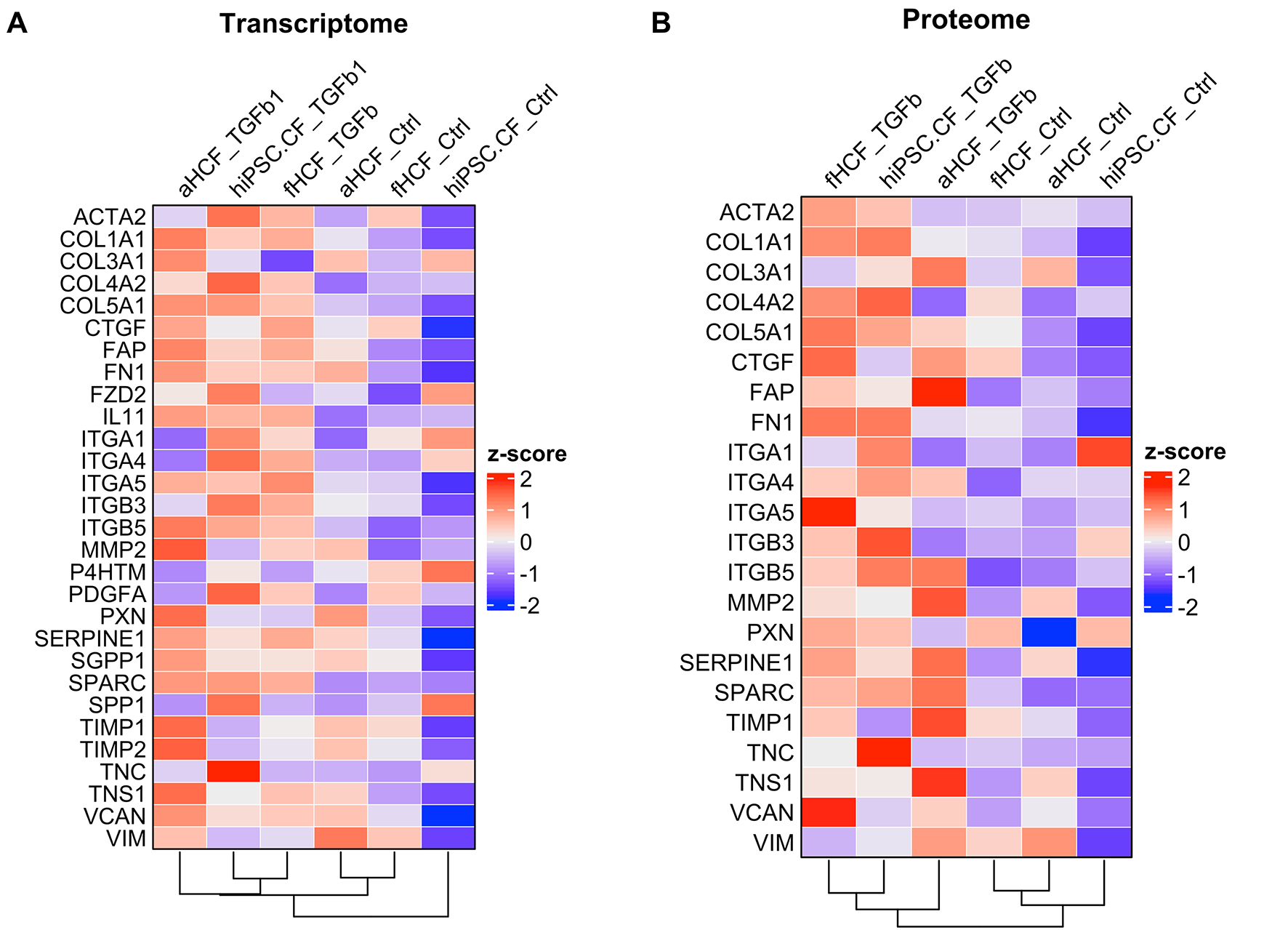

Supplement: Supplementary file 5 [file Image_4.TIF]

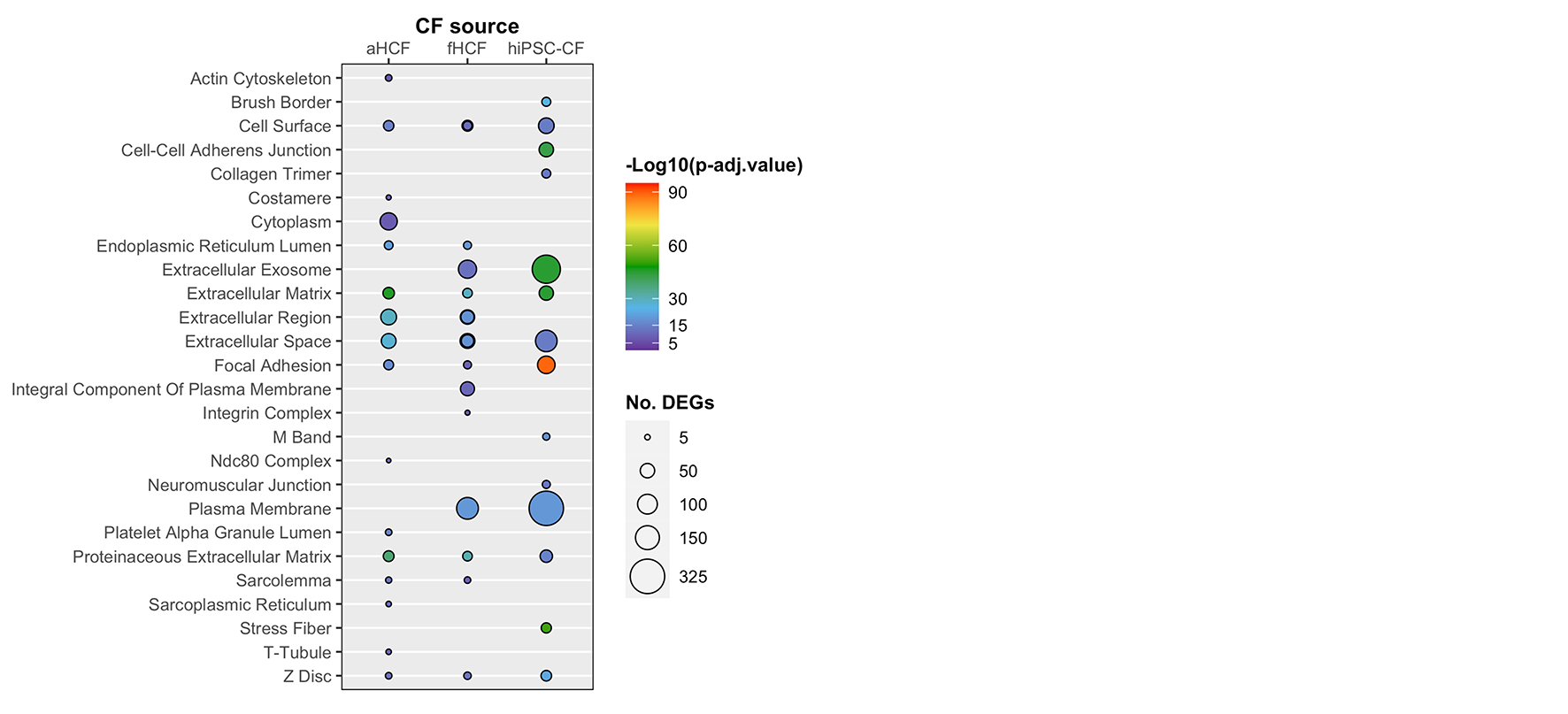

Supplement: Supplementary file 6 [file Image_5.TIF]

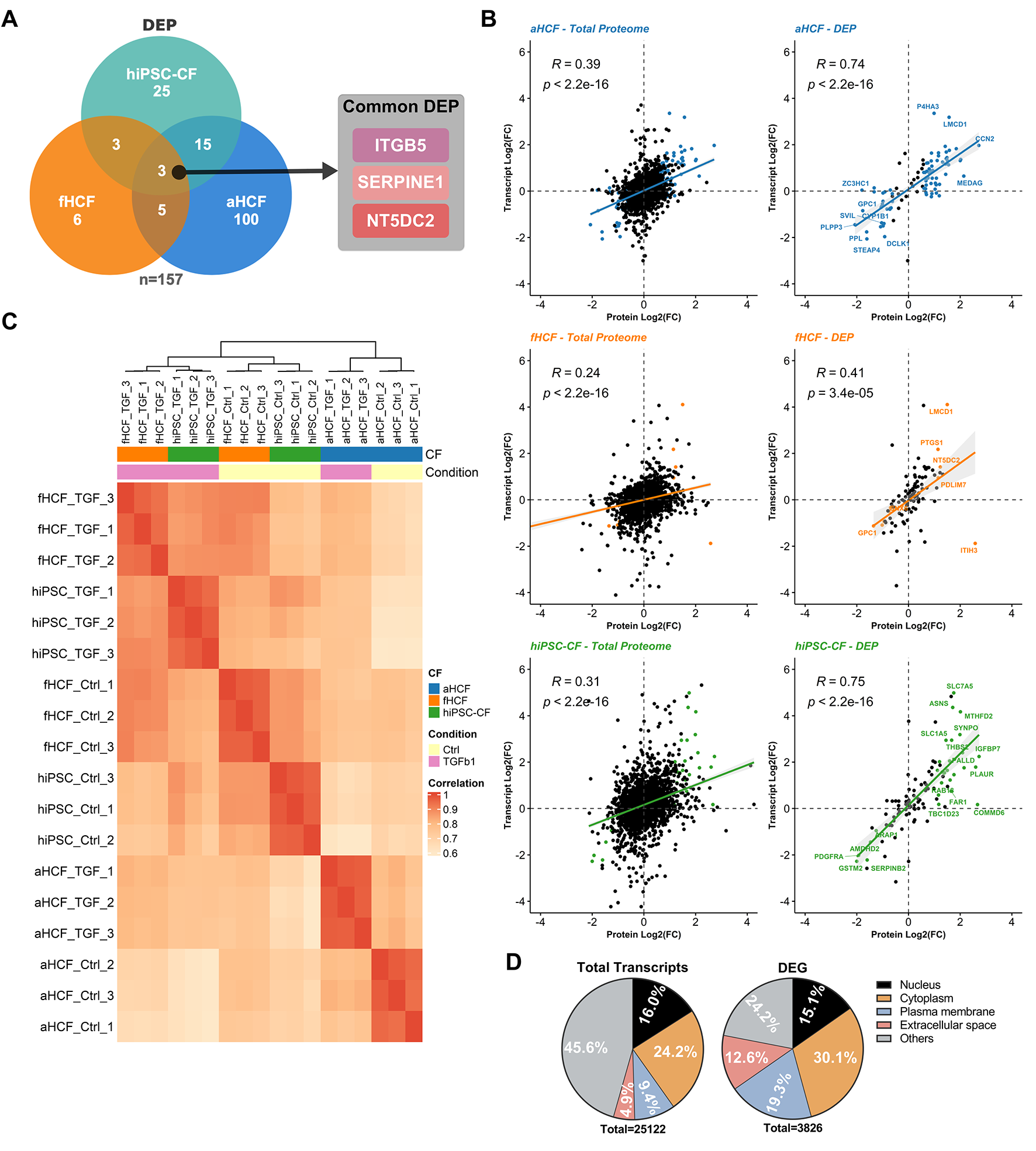

Supplement: Supplementary file 7 [file Image_6.TIFF]

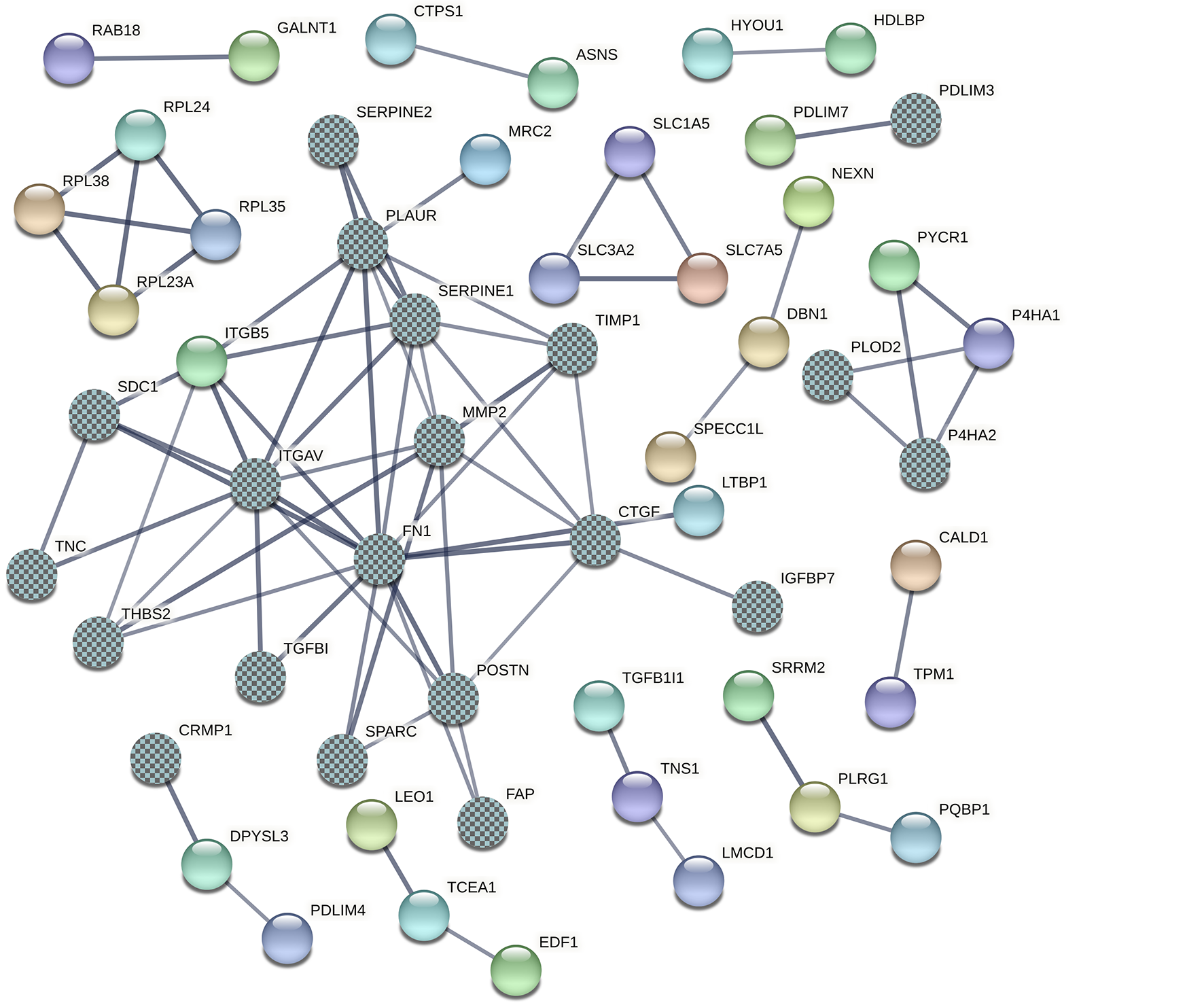

Supplement: Supplementary file 8 [file Image_7.TIFF]

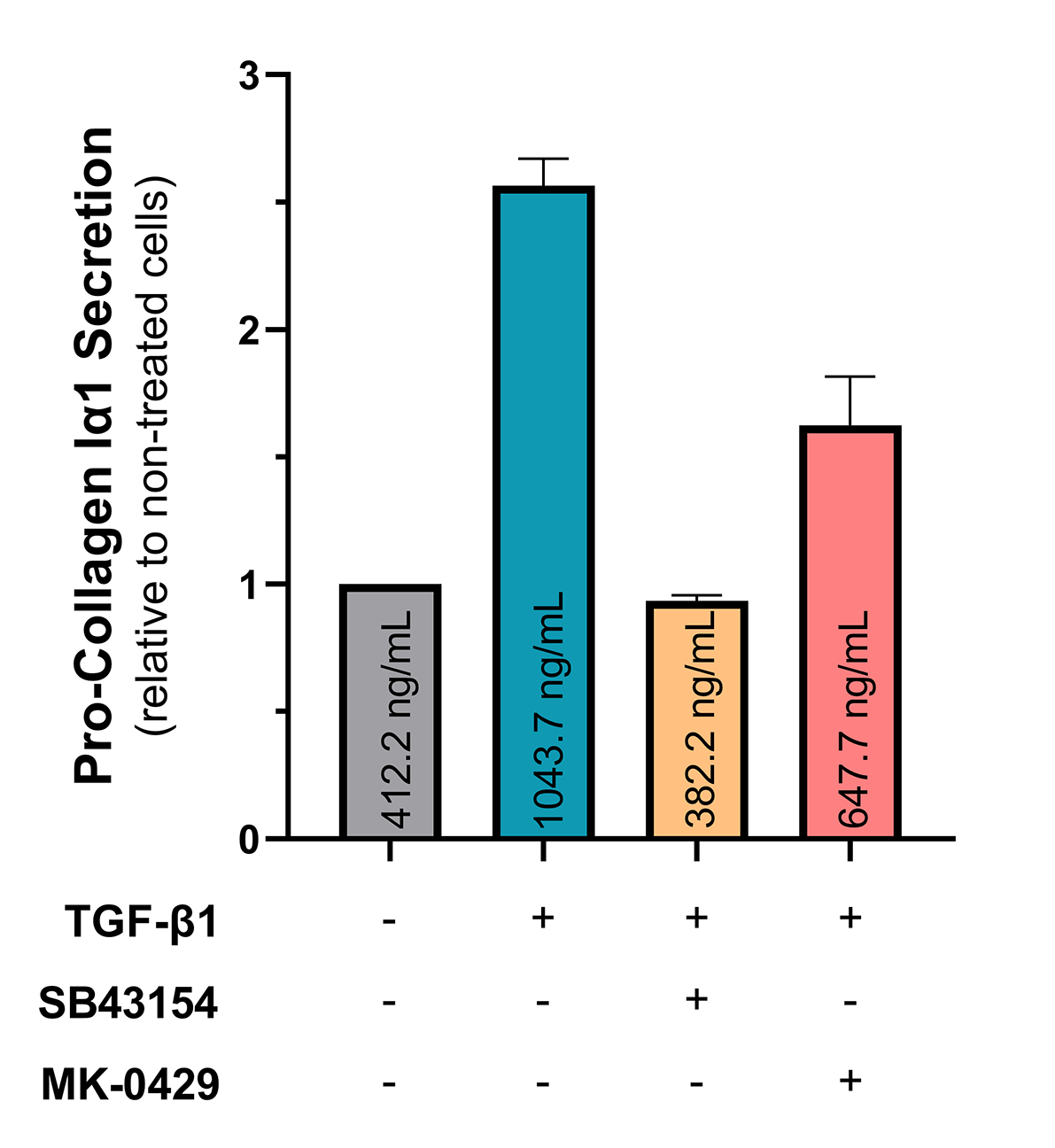

Supplement: Supplementary file 9 [file Image_8.TIF]
